# Latent profile analysis of adaptation types and stress among medical students during the COVID-19 pandemic in South Korea

**DOI:** 10.12669/pjms.39.1.7196

**Published:** 2023

**Authors:** Kwi Hwa Park, Sun Ju Im, Sun Young Kyung, So Jung Yune

**Affiliations:** 1 Kwi Hwa Park, Department of Medical Education, Gachon University College of Medicine Incheon, Rep. of Korea; 2 Sun Ju Im, Department of Medical Education, Pusan National University School of Medicine Busan, Rep. of Korea; 3 Sun Young Kyung, Department of Internal Medicine, Gachon University College of Medicine Incheon, Rep. of Korea; 4 So Jung Yune, Department of Medical Education, Pusan National University School of Medicine Busan, Rep. of Korea

**Keywords:** COVID-19, Coping strategies, Medical students, Occupational stress, Personality traits

## Abstract

**Objective:**

This study aimed to identify profile groups based on personality traits and coping strategies exhibited by medical students in the context of COVID-19.

**Methods:**

We conducted a cross-sectional survey and latent profile analysis to investigate differences in stressors, psychological distress, and stress levels with academic variables. We collected data online (Google survey form) in November and December 2021. The participants included a total of 260 1^st^ and 2^nd^ year medical students, all completed questionnaires containing the following sections: Big Five Inventory, coping strategies, COVID-19 stressors, Kessler Psychological Distress Scale, and stress level with academic variables. For analysis, a latent profile analysis, ANOVA, and χ2 were used.

**Results:**

The results reveal the three following profile groups: adaptive (lowest neuroticism, low mental disengagement), middle-adaptive (moderate neuroticism, low mental disengagement), and maladaptive (highest neuroticism and mental disengagement), respectively comprising 25.0%, 39.2%, and 35.8% of the study sample. There were no statistically significant intergroup differences regarding grade (χ2=3.345, p=0.188) or gender (χ2=1.197, p=0.550). The maladaptive group was strongly associated with perceived stress during the COVID-19 pandemic (p<0.001).

**Conclusion:**

These findings highlight the value of considering profile groups when determining whether students require additional support during pandemics.

## INTRODUCTION

COVID-19 was first identified toward the end of December 2019.^1^ Since then, the disease has quickly spread worldwide. The prolonged nature of the subsequent pandemic has induced substantial fatigue leading to fear, stress, and anxiety in daily life, thus affecting the psychological health of many individuals.^2^,^3^ Medical students face the same issues. The pandemic has induced restrictions on most activities at medical schools, including lectures and assessments, which have not been available in the face-to-face format.^4^ Along with these changes in traditional teaching practices, students have lost opportunities for peer interactions and social connectedness.^5^ In fact, studies have shown higher rates of pandemic-related anxiety and depression among medical students, including more fragility than among non-medical students.^6^,^7^ This increased stress can affect physical health, create psychological difficulties, and is associated with academic performance and adjustment factors.^8^ Thus, it is essential to ensure that medical students receive adequate mental health support during COVID-19.

Even in a crisis such as the COVID-19 pandemic, some individuals remain unperturbed, while others develop negative perceptions.^9^ The starting point for explaining these behavioral differences is personality, which is a stable pattern of thinking, feeling, and behaving when coping with environmental stimuli through interactions between the individual and their environment.^10^ Such traits are most often evaluated using the Five-Factor Model (FFM).^11^ Recent studies have investigated the relationship between personality traits and individual responses to the COVID-19 pandemic, showing that higher neuroticism is associated with more negative affective responses and perceived stress.^9^,^12^,^13^ Although these studies have targeted various demographics, including healthcare workers,^12^ general adults,^9^ and college students,^14^ there is still a lack of pandemic-related evidence on personality traits and perceived stress/affective responses in medical students.

As with personality traits, coping styles are associated with different individual approaches to crises and play crucial roles in stress responses.^15^ Specifically, coping style refers to the cognitive and behavioral strategy that an individual may use to manage perceived internal and external demands when placed in a stressful situation.^16^ A given coping style may be positive (e.g., managing problems, finding solutions, and quickly adjusting to stressors) or harmful (e.g., avoidance and social isolation). Here, it is essential to note that positive coping is associated with less stressful experiences and better psychological adaptation. Thus, the individual coping style may variously mediate the process of stress relief during crises such as pandemics.^17^

However, previous studies have thus far applied variable-oriented approaches. It is not suitable for understanding various combinations that exist in the relationships between variables.^18^ According to previous studies, personality traits and coping styles are interrelated and associated with perceived differences in crises.^19^ To address the gap in the literature, this study conducted a person-centered analysis of personality traits and coping styles through a latent profile analysis (LPA), an established person-oriented method.^20^ In this regard, we aimed to identify potential profiles among medical students based on the scoring probability of individual personality traits and coping styles. We know of no previous studies that have employed LPA to investigate personality traits and coping styles in medical students with a focus on perceived stress during COVID-19. In this context, this study used the LPA approach to identify the profiles of medical students according to their personality traits and coping strategies. We then analyzed those profiles to assess differences in COVID-19 stressors, psychological distress, and stress level with academic variables.

## METHODS

This study collected data through a cross-sectional survey among medical students. The study participants included a total of 260 medical students from two schools; that is, 138 (53.1%) in their first year and 122 (46.9%) in their 2^nd^ year, including 165 males (63.5%) and 95 females (36.5%).

### Instruments Big Five Inventory of personality traits:

We investigated personality traits using the Korean Short Version of the Big Five Inventory, which was developed by Kim et al.^21^ The tool consists of 15 items that are equally divided across the five subfactors of Openness, Conscientiousness, Neuroticism, Extraversion, and Agreeableness, each of which have shown good reliability based on Cronbach’s alpha values of 0.894, 0.767, 0.823, 0.602, and 0.735, respectively.

### Coping strategies:

We assessed coping strategies in stressful situations using tools from Savitsky et al.^22^ While the original scale consisted of 12 items across five subfactors, this study used eight items across three subfactors after conducting a factor analysis. Specifically, this included resilience (four items), seeking information and consultation (two items), and mental disengagement (two items), each of which has shown good reliability based on Cronbach’s alpha values of 0.726, 0.665 and 0.648, respectively.

### COVID-19 stressors:

We measured COVID-19 stressors using the tool from Park et al.,^23^ wherein individuals rate each stress level against 23 stressors over the previous week in the context of COVID-19. The tool contains three subfactors, including infection-related (eight items), activity-related (seven items), and financial/resource-related (five items), each of which has shown good reliability based on Cronbach’s alpha values of 0.867, 0.863, and 0.886, respectively.

### Kessler Psychological Distress Scale:

We measured psychological distress using the 10-item Kessler Psychological Distress Scale developed by Kessler et al.^24^ The tool measures the degree of distress based on questions about anxiety and depressive symptoms that individuals have experienced over the previous four weeks of COVID-19. The tool has shown good reliability based on Cronbach’s alpha value of 0.957.

### Stress level with academic variables:

We investigated stress levels using the 11-item tool from Abdulghani et al.,^25^ which measures student stress levels to academic variables (e.g., online learning and lectures) in COVID-19. The scale has shown good reliability based on Cronbach’s alpha value of 0.953.

### Data collection:

We collected data online (Google survey form) in November and December 2021. To ensure ethical protection for participants, we did not include personally identifiable information in the survey guideline. We specify the study purpose and contents and also guarantee their anonymity. This study was approved by the Gil Medical Center Institutional Review Board of Gachon University (IRB approval no., GCIRB-2021-447).

### Statistical analysis:

We conducted the LPA statistical analysis with the Mplus software for Windows (Version 8.7; Muthen & Muthen, 1998-2021),^26^ thus classifying participants into distinct profiles, with the mean scores of their personality traits and coping strategies used as LPA indicators. We adopted three information criteria to determine the optimal profile model for the data, including the Akaike Information Criterion (AIC), Bayesian Information Criterion (BIC), and Sample-size Adjusted BIC (aBIC), with smaller values indicating better model fit in each case. We also implemented the bootstrap likelihood ratio test (BLRT) and Lo–Mendel–Rubin adjusted likelihood ratio test (LMR). We examined entropy values, in which those closer to 1.0 indicated better classification precision. Moreover, we considered other factors determining the optimal number of profiles in conjunction with the percentage of participants per class (more than 5%), theoretical justification, and interpretability.^27^ After determining the number of latent classes according to these indices, we conducted the χ2 test and a one-way ANOVA.

## RESULTS

The LPA fit indices for profile models two through five. Table-I The AIC, BIC, and aBIC values decreased with an increasing number of classification profiles, from 2-class to 5-class. The 2-class had the largest AIC, BIC, and aBIC values, indicating that it was worse for the data than others. The 3-class to 5-class models were marked with sufficient entropy values above 0.80. Looking at the 3-class and 4-class models, the entropy value of the 4-class was higher, but this model was not appropriate because the class size was less than 5%. Thus, the 3-class model was better than the 4-class model. The 4-class to 5-class models were not selected due to nonsignificant LMR. After careful consideration, we selected the 3-class model as the optimal number of profiles (Table-I).

**Table-I:** Fitness indicators of latent profile models (N=260).

No. of Profiles	AIC	BIC	aBIC	Entropy	LMR (*p*)	BLRT (*p*)	Latent class size (%)				

							1	2	3	4	5
2	4871.425	4960.442	4881.182	0.733	0.001	0.001	45.4	54.6			
3	4728.914	4849.977	4742.184	0.819	0.001	0.001	39.2	35.8	25.0		
4	4694.684	4847.793	4711.465	0.853	0.489	0.001	35.8	25.0	35.3	3.9	
5	4680.465	4865.621	4700.76	0.814	0.169	0.001	33.1	25.8	3.5	13.4	24.2

***Note:*** AIC = Akaike’s information criterion; BIC = Bayesian information criterion; aBIC = adjusted Bayesian information criterion; BLRT = bootstrap likelihood ratio test; LMR = Lo-Mendell-Rubin adjusted likelihood ratio test.

**Fig.1:**
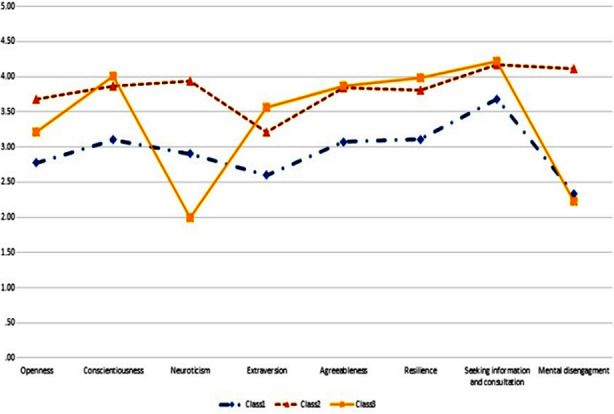
Differences in personality traits and coping strategies of each group.

We categorized the participants into three groups, including the adaptive, middle-adaptive, and maladaptive groups, which comprised 25.0%, 39.2%, and 35.8% of the sample, respectively (Table-II, Fig.1). Three groups were named based on their adaptability to medical school academic situations based on their personality traits and coping strategies. Profile-1 was the middle-adaptive group, demonstrating average neuroticism in personality and low mental disengagement in coping strategies. Profile-2 was the maladaptive group, which showed the highest levels of neuroticism and mental disengagement. Profile-3 was the adaptive group, which showed the lowest levels of neuroticism and low mental disengagement (Table-II, Fig.1).

**Table-II:** Means of the three latent profiles (N=260)

Profile	N	%	Mean score							

			O	C	N	E	A	R	SIC	MD
1	102	39.2	2.78	3.10	2.90	2.60	3.08	3.11	3.68	2.33
2	93	35.8	3.68	3.86	3.94	3.21	3.84	3.81	4.17	4.12
3	65	25.0	3.22	4.01	1.99	3.56	3.87	3.98	4.22	2.23

Total	260	100	3.21	3.60	3.05	3.06	3.55	3.58	3.99	2.94

***Note:*** O = Openness; C = Conscientiousness; N = Neuroticism; E = Extraversion; A = Agreeableness; R = Resilience; SIC = seeking information and consultation; MD = mental disengagement.

### Demographic characteristic differences in the identified profiles:

The demographic characteristics associated with the three profiles are listed in Table-III. There were no statistically significant differences in grade (χ2=3.345, p=0.188) or gender between these profiles (*χ2*=1.197, *p*=0.550). (Table-III)

**Table-III:** Demographic characteristics associated with the three identified profiles.

Profile	Year		Gender	

	1	2	Male	Female
1	61	41	63	39
	44.2%	33.6%	38.2%	41.1%
2	47	46	63	30
	34.1%	37.7%	38.2%	31.6%
3	30	35	39	26
	21.7%	28.7%	23.6%	27.4%
	χ2=3.345, p=0.188		χ2=1.197, p=0.550	

The results of the three profile comparisons for stressors, psychological distress, and stress level with academic variables in COVID-19 are listed in Tale-IV. As for the sub-factors of COVID-19 stressors, we found significant differences regarding infection-related (*F*=62.221, *p*<0.001), activity-related (*F*=68.251, *p*<0.001), and financial/resource-related (*F*=51.199, *p*<0.001). Profile-2 (maladaptive group) was the highest, while profile-3 (adaptive group) was the lowest.

Psychological distress was also significantly different between the three profiles (*F*=149.511, *p*<.001), with Profile-2 showing the highest mean scores. Finally, profile 2 also had significantly higher stress levels with academic variables when compared to the other profiles (*F*=112.932, *p*<0.001). In the context of COVID-19, the maladaptive group was, therefore, more stressed about academic variables (e.g., online lectures and learning) (Table-IV).

**Table-IV:** Associations between profiles and variables.

	Profile	N	M	SD	F	P	Scheffe
***COVID-19 stressors***							
Infection-related	1	102	2.99	.69	62.221	<0.001	2>1, 2>3
	2	93	3.97	.58			
	3	65	2.93	.85			
Activity-related	1	102	3.07	.68	68.251	<0.001	2>1, 2>3
	2	93	4.11	.57			
	3	65	3.15	.77			
Financial/ resource-related	1	102	2.73	.83	51.199	<0.001	2>1, 2>3
	2	93	3.91	.92			
	3	65	2.75	.97			
Psychological distress	1	102	2.18	.71	149.511	<0.001	2>1, 2>3
	2	93	3.82	.93			
	3	65	1.87	.71			
Stress level with academic variables	1	102	2.41	.79	112.932	<0.001	2>1, 2>3
	2	93	4.03	1.02			
	3	65	2.11	.89			

## DISCUSSION

Working in the COVID-19 context, this study conducted an LPA to examine the profiles of medical students based on their personality traits and coping strategies. We also explored differences between the profiles thus identified, with a focus on COVID-19 stressors, psychological distress, and stress level with academic variables. In sum, the LPA revealed three distinct groups, including adaptive (lowest neuroticism, low mental disengagement), middle-adaptive (moderate neuroticism, low mental disengagement), and maladaptive (highest neuroticism and mental disengagement), which comprised 25.0%, 39.2%, and 35.8% of the study sample, respectively. Given the percentage of individuals in the maladaptive group, our analyses suggest that neuroticism and mental disengagement coping strategies may be expected in medical students.

Such individuals may engage in habits such as overeating to calm themselves and/or use alcohol or sedatives to feel better. Students with neuroticism have shown high rates of avoidant coping mechanisms, such as eating, playing video games, and shopping,^19^,^28^ thus indicating a relationship between neurotic personality traits and negative coping strategies. Individuals with high neuroticism are also more likely to choose negative coping strategies, such as mental disengagement. In this regard, the higher proportion of participants in the maladaptive (vs. adaptive) group indicates that many may be vulnerable to unexpected crises. However, medical students are less likely to ask for support,^29^ which highlights the importance of screening procedures aimed at maladaptive profile traits, thus ensuring early identification and priority support.

Therefore, of the three groups, we need to pay more attention to the maladaptive group. It is because the maladapted group has high levels of neuroticism and mental disengagement. The educational situation in medical school can cause high academic stress. In addition, in crises such as COVID-19, high neuroticism may experience more negative emotions or stress than other groups of students.^9^,^12^,^13^ Therefore, it is necessary to carefully examine the students’ psychological state in the maladaptive group, provide student support programs such as counseling and meditation, and train positive coping strategies.

Of note, there were no significant differences in gender or grade between groups, suggesting even distributions between the adaptive, maladaptive, and middle-adaptive profiles. Therefore, intervention programs should be based on profile traits, not grade or gender. We found that the maladaptive group was strongly associated with perceived stress during the pandemic. Specifically, this group responded more negatively to infection-related, activity-related, and financial/resource-related stressors caused by COVID-19. They also showed significantly higher psychological distress. To their academic variables, the stress level for online classes showed the same results. These findings are supported by many previous studies conducted during the pandemic.^5^,^12^,^14^,^30^

This study has several important implications. For one, it was the first to conduct an LPA to investigate potential profile groups among medical students based on the Big Five personality traits and coping strategies in the COVID-19 context. Moreover, our person-centered design distinguishes our research from other relevant studies which have implemented variable-centered approaches. We also found a higher proportion of participants in the maladaptive (vs. adaptive) group, including higher stress levels, emphasizing the importance of advanced screening for medical students. Our findings indicate the need for increased awareness and sensitivity toward their mental health, especially during COVID-19.

By extension, this study contributes to a deeper understanding of how personality traits and coping strategies impact individual behaviors during crises such as pandemics. Our findings may therefore provide guidance when attempting to screen psychologically vulnerable medical students. These results should help medical school administrators and educators provide students with adequate resources, including counseling, peer advocacy, and support.

### Limitations:

First, we solely investigated medical students from two schools in South Korea, which limits generalizability. Second, we did not recruit clinical medical students in their third and fourth years. Future studies should therefore analyze the stability of the latent profiles and their differences across all grades. Third, we relied on self-reported measures. Here, self-presentation bias is known to exist in self-reported data, such as those about personality factors. This highlights the need for additional methods, including qualitative analyses, to support the validity of the results.

## CONCLUSION

This study demonstrated the importance of using profile groups based on personality traits and coping strategies when considering how students are influenced by stress during COVID-19. In our sample, more medical students were in the maladaptive group (highest neuroticism and mental disengagement) compared to the adaptive group. Of note, the maladaptive group showed the strongest association with perceived stress. Thus, our findings highlight the value of considering these profile groups when determining whether students require additional support measures, especially during crises such as the current pandemic.

### Authors’ Contribution:

**KHP:** did conceptualization, data curation, formal analysis, methodology, and writing-original draft.

**SJI:** did the investigation, formal analysis, writing review, and editing.

**SYK:** did conceptualization, investigation, formal analysis, methodology, writing review, and editing.

**SJY:** did conceptualization, data curation, writing review & editing, final approval of the manuscript, and supervision. She is also responsible for the accuracy and integrity if the manuscript.
